# Initiating antiretroviral therapy within 2 weeks of anti-*Pneumocystis* treatment does not increase mortality or AIDS-defining events in patients with HIV-associated moderate to severe *Pneumocystis* pneumonia: results of a prospective observational multicenter study

**DOI:** 10.1186/s12890-022-02118-4

**Published:** 2022-08-25

**Authors:** Yan-Ming Zeng, Yao Li, Yan-Qiu Lu, Min Liu, Jing-Min Nie, Jing Yuan, Vijay Harypursat, Yi-Hong Zhou, Yuan-Yuan Qin, Xiao-Hong Chen, Yu-Lin Zhang, De-Fa Zhang, Ni Wang, Hui Chen, Qun Tian, Yang Zhou, Ying-Mei Qin, Xin-Ping Yang, Yao-Kai Chen

**Affiliations:** 1 Division of Infectious Diseases, Chongqing Public Health Medical Center, 109 Baoyu Road, Shapingba District, Chongqing, 400036 China; 2 Department of Infectious Diseases, The Fourth Affiliated Hospital of Harbin Medical University, Harbin, China; 3 Division of Infectious Diseases, Beijing Youan Hospital, Capital Medical University, Beijing, China; 4 Department of Infectious Diseases, Tianjin Second People’s Hospital, Nankai University, Tianjin, China; 5 School of Biomedical Engineering, Capital Medical University, Beijing, China; 6 Division of Infectious Disease, The Third People’s Hospital of Guilin, Guangxi, China; 7 Division of Infectious Diseases, Shenzhen Third People’s Hospital, Shenzhen, China; 8 Division of Infectious Diseases, The Fourth People’s Hospital of Nanning, Guangxi, China; 9 Division of Infectious Disease, Yunnan Provincial Infectious Disease Hospital, Yunnan, China

**Keywords:** HIV, Pneumocystis pneumonia, Antiretroviral therapy, Initiation

## Abstract

**Background:**

The mortality rate remains high among patients with coinfection with *Pneumocystis* pneumonia (PCP) and HIV. The timing for initiation of antiretroviral therapy (ART) after a diagnosis of moderate to severe PCP remains controversial, however. We therefore designed the present study to determine the optimal timing for ART initiation in AIDS-associated PCP (AIDS/PCP) patients.

**Methods:**

This was a multicenter, observational, prospective clinical trial. Eligible participants were recruited from 14 hospitals in mainland China, and assigned to an Early ART arm (initiation of ART ≤ 14 days after PCP diagnosis) and a Deferred ART arm (initiation of ART > 14 days after PCP diagnosis). The primary outcomes were death and the incidence of AIDS-defining events at week 48. The secondary outcomes were the changes in CD4+ T-cell counts from baseline values at weeks 12, 24, and 48, the virological suppression rate at week 24 and week 48, the rate of development of PCP-associated immune reconstitution inflammatory syndrome (PCP/IRIS), and the rate of adverse events over 48 weeks.

**Results:**

The present study was performed using the data of 363 participants, with 169 participants in the Early ART arm, and 194 participants in the Deferred ART arm. Immunological and virological outcomes were found to be similar in both treatment arms. At week 48, there were no significant differences for the incidence of mortality (20 vs. 26, *p* = 0.860), and AIDS-defining events (17 vs. 26, *p* = 0.412). Over 48 weeks, the rates of PCP/IRIS (2 vs. 3, *p* = 1.000), adverse events (70 vs. 72, *p* = 0.465), and grade 3 or 4 adverse events (28 vs. 34, *p* = 0.919) did not reach statistical significance. A significant difference observed between two study arms was that 11 participants (55.0%) in the Early ART arm compared to 23 participants (88.5%) in the Deferred ART arm (*p* = 0.026) succumbed before ART had ever been started.

**Conclusions:**

Early ART initiation results in no increase in mortality, AIDS-defining events, IRIS, adverse events, and immunological or virological outcomes. These results support the early initiation of ART in patients with moderate to severe AIDS/PCP.

*Clinical trial registration*

The present trial was registered at Chinese Clinical Trial Registry (ChiCTR1900021195). Registered 1 February 2019, http://www.chictr.org.cn/showproj.aspx?proj=35362.

## Introduction

*Pneumocystis* pneumonia (PCP) is caused by a yeast-like fungus, *Pneumocystis jirovecii*, and is a commonly occurring opportunistic infection (OI) in HIV-infected patients [1, 2]. The incidence of PCP increased rapidly during and after the 1980s, and is currently as high as 75% in HIV-infected patients, especially in those with CD4+ T-cell counts < 200 cells/μL, and results in mortality in up to 40% of patients [3]. Since the mid-1990s, the widespread use of effective PCP prophylaxis and early antiretroviral therapy (ART) has led to a substantial decline in the prevalence of PCP among HIV-infected patients [4].

AIDS-associated PCP remains, however, the predominant OI among AIDS patients [5]. One past study reported that in China the prevalence of PCP in HIV-infected patients who have not initiated ART was 22.4% [5]. The clinical course of treated PCP is associated with 20–40% mortality in individuals with profound immunosuppression [6]. In addition, a cohort study conducted in China from 2009 to 2018, showed that in-hospital mortality among HIV-infected patients who had concomitant PCP remained unacceptably high, at 17.3% (173/1001) [7]. PCP thus remains a life-threatening OI, and is the most common cause of death in HIV-infected patients with severe immunosuppression [6].

The United States Department of Health and Human Services (DHHS) guidelines [6] recommend that ART should be initiated within 2 weeks of diagnosis of PCP (AI), and this recommendation is supported by the results of a randomized controlled trial conducted from 2003 to 2006 in Puerto Rico, South Africa, and the United States, which included 282 HIV-infected patients with OIs other than TB [8]. The Chinese guidelines for the diagnosis and treatment of HIV/AIDS (2018) also recommends initiating ART within 2 weeks of treatment of PCP [9]. Nonetheless, there remains no recent local high-quality evidence in China to support this recommendation. Therefore, we conducted the present controlled trial, which aimed to investigate the timing of ART initiation in AIDS patients with moderate to severe PCP in China.

## Methods

### Study design

This study was a multicenter, observational, prospective clinical trial. Eligible participants were recruited from 14 hospitals in China. Forty-eight weeks of follow-up during this study was scheduled at weeks 12, 24, and 48. Participants were assigned to either the Early ART arm (initiation of ART ≤ 14 days after PCP diagnosis) or the Deferred ART arm (initiation of ART > 14 days after PCP diagnosis).

### Participants

Participants admitted to hospital from March 2019 to October 2020 at the following 14 hospitals in mainland China were assessed for eligibility: Chongqing Public Health Medical Center, Beijing Youan Hospital of Capital Medical University, the Fourth Affiliated Hospital of Harbin Medical University, the Second People's Hospital of Tianjin, the First Hospital of Changsha, Liuzhou General Hospital, the Third People's Hospital of Guilin, the Third People’s Hospital of Shenzhen, Guiyang Public Health Clinical Center, the Third People’s Hospital of Kunming, Yunnan Provincial Infectious Disease Hospital, the Fourth People’s Hospital of Nanning, Guangxi Longtan Hospital, and Xixi Hospital of Hangzhou.

### Diagnostic criteria

Participants had to meet the following criteria [6, 9] for a diagnosis of moderate to severe PCP:

1. Progressive dyspnea, fever, and non-productive cough for days or weeks;
2. Diffuse “ground glass” interstitial infiltrates spreading from the pulmonary hilum on a chest radiograph.
3. Alveolar-arterial O2 gradient, (A-a) DO_2_ ≥ 35 mmHg, or room air arterial oxygen, partial arterial oxygen pressure (PaO_2_) < 70 mmHg.
4. Serum beta-1,3-glucan levels higher than normal.
5. Microbiological confirmation by positive PCR and/or Grocott’s methenamine silver (GMS) results for sputum, pulmonary aspirate, or bronchoalveolar lavage fluid (BALF) samples.

All the enrolled patients either met criteria (1), (2), and (3) (presumptive diagnosis), or criteria (1), (2), (3), and (5) (confirmative diagnosis), with or without criterion (4).

We defined PCP/immune reconstitution inflammatory syndrome (PCP/IRIS) as occurring when the subject experiences a paradoxical exacerbation of either clinical symptoms or radiological signs of PCP after the initiation of ART despite receiving appropriate drug treatment for PCP.

### Inclusion criteria

Eligible participants were required to satisfy the following inclusion criteria:

1. 18 years or older.
2. diagnosis of confirmed HIV-1 infection and presumptive/definitive moderate to severe PCP.
3. ART-naïve.
4. willing to sign informed consent.

### Exclusion criteria

Participants were excluded if they:

1. were intolerant to any of the interventions.
2. had a hemoglobin level < 60 g/L, white blood cell count < 1.0 × 10^9^/L, neutrophil count < 0.5 × 10^9^/L, platelet count < 50 × 10^9^/L, blood amylase > 2 × upper normal limit (UNL), serum creatinine > 1.5 × UNL, aspartate aminotransferase/alanine aminotransferase (AST/ALT) > 5 × UNL, total bilirubin > 2 × UNL, serum creatine phosphokinase > 2 × UNL.
3. had concomitant other unstable OIs.
4. had serious heart, brain, lung, kidney, tumor, or other systemic diseases.
5. were pregnant or breastfeeding women.
6. had a severe mental illness.
7. were intravenous recreational drug users.
8. were not of Chinese nationality.

### Interventions

For the treatment of moderate to severe PCP, a conventional treatment regimen was given to all participants, in accordance with the recommendations of current Chinese guidelines for the diagnosis and treatment of HIV/AIDS [9]. Trimethoprim-sulfamethoxazole (TMP-SMZ) combined with prednisone was thus the preferred treatment regimen for moderate to severe PCP. An alternative regimen may have been used if the preferred regimen was not tolerated, or if the patient was allergic to the preferred regimen. Clindamycin or caspofungin could potentially be used as alternative therapeutic drugs. All the enrolled participants received HIV treatment regimens in accordance with the recommendations of the Chinese guidelines for the diagnosis and treatment of HIV/AIDS [9].

### Follow-up

Participants were assessed in person at baseline, week 12, week 24, and week 48. CD4+ T-cell counts were performed whenever possible at each follow-up visit. HIV RNA viral loads were reviewed in participants at baseline, week 24, and week 48.

### Study outcomes

The primary outcomes were death from any cause, and the incidence of an AIDS-defining event at week 48, which was defined as any new or relapsing HIV-related OI.

Secondary outcomes were as follows: (A) the changes in CD4+ T-cell counts from baseline and at weeks 12, 24, 48; (B) virological suppression rate at week 24 and week 48, which was defined as the HIV RNA viral load of a patient below the lower limit of detection of currently-used HIV RNA assays (50 RNA copies/mL); (C) rates of development of PCP/IRIS, which was defined as a paradoxical exacerbation of either clinical symptoms or radiological signs of PCP after the initiation of ART, despite receiving appropriate drug treatment for PCP; (D) rates of admission to Intensive Care Unit (ICU); (E) rates of requirement for intubation; (F) adverse events over 48 weeks.

### Data analysis

Baseline characteristics, including age, gender, CD4+ T-cell counts, HIV RNA viral load, beta-1,3-glucan, and other characteristics, were compared between the Early ART arm and Deferred ART arm. Categorical variables were analyzed via the Chi-squared (χ^2^) test, continuity corrections, or Fisher’s exact test. Continuous variables were analyzed via Wilcoxon tests, and we applied the median (IQR) to data. Interactions with prognostic factors were also examined with the Kaplan–Meier method and the Cox proportional-hazards model. Multivariable analyses were used to estimate the simultaneous effects of prognostic factors on survival. A *p* value of less than 0.05 was considered statistically significant. All analyses were performed using the Statistical Package for the Social Sciences (SPSS), Version 25.0 (IBM-SPSS, Armonk, New York, USA).

## Results

### Participants

**Fig. 1:**
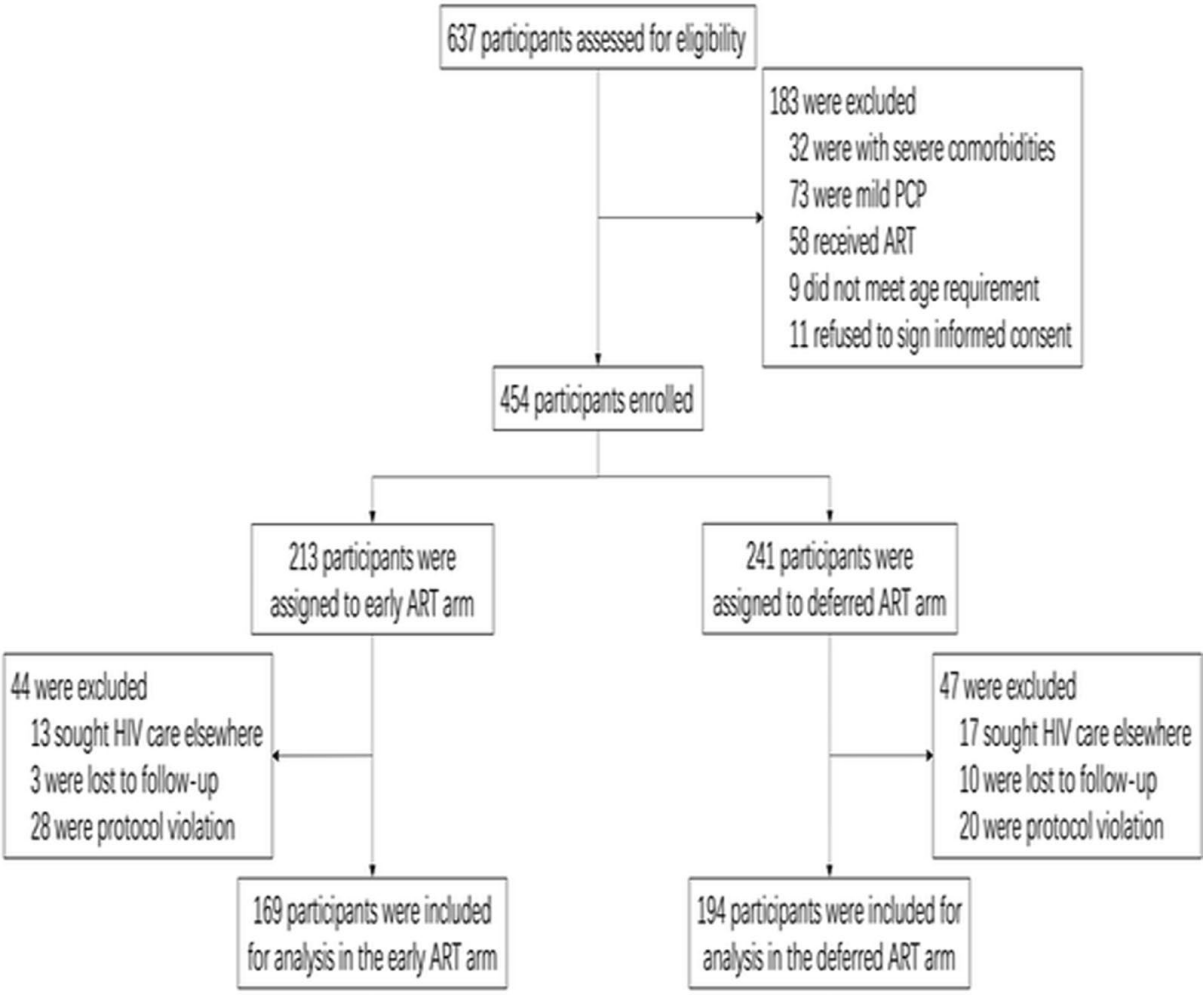
Patient enrollment and treatment assignment

The present study was performed with 363 participants for analysis, with 169 participants in the Early ART initiation arm, and 194 participants in the Deferred ART initiation arm. Patient enrollment and treatment assignment is shown in Fig. 1. Gender, ethnic group, alcohol use, body mass index (BMI), respiratory rate, temperature, platelets, white blood cell count, alanine aminotransferase (ALT), aspartate aminotransferase (AST), total bilirubin, creatinine, blood urea nitrogen (BUN), PaO_2_, beta-1,3-glucan, CD4+ T-cell count, CD4/CD8 ratio, and log_10_ HIV-1 RNA copies at baseline showed no significant differences in both arms. However, there were significant differences in age, smoking, and red blood cell counts, as seen in Table 1.

**Table 1:** Demographics and clinical baseline characteristics

Characteristics	Total (n = 363)	Early ART arm (n = 169)	Deferred ART arm (n = 194)	*p*-value
*Demographic characteristics*				
Male, n (%)	296 (81.5)	138 (81.7)	158 (81.4)	1.000
Ethnic group, Han, n (%)	345 (95.0)	160 (94.7)	185 (95.4)	0.954
Age, (years, IQR)	47.0 (34.0, 55.0)	44.0 (34.0, 52.5)	48.5 (35.0, 56.0)	0.034
Smoking, n (%)	142 (39.1)	50 (29.6)	92 (47.4)	0.001
Alcohol use, n (%)	94 (25.9)	38 (22.5)	56 (28.9)	0.206
BMI, (kg/m^2^, IQR)	20.2 (18.7, 22.5)	20.2 (18.7, 22.9)	20.2 (18.6, 22.3)	0.780
Respiration, (times/min, IQR)	22 (20, 25)	22 (20, 25)	22 (21, 24)	0.715
Temperature, (℃, IQR)	37.0 (36.5, 38.0)	37.0 (36.6, 38.0)	37.0 (36.5, 38.0)	0.906
*Laboratory results*				
Red blood cell count (× 10^9^ /L, IQR)	4.0 (3.6, 4.4)	4.1 (3.7, 4.5)	3.9 (3.5, 4.3)	0.022
Platelets (× 10^9^/L, IQR)	228.0 (171.0, 287.0)	224.0 (173.0, 287.5)	231.0 (169.8, 286.5)	0.644
White blood cell count (× 10^9^ /L, IQR)	5.6 (4.1, 7.8)	5.7 (4.0, 8.2)	5.4 (4.3, 7.2)	0.474
ALT (U/L, IQR)	24 (15, 38)	24 (14, 44)	22 (15, 37)	0.303
AST (U/L, IQR)	35 (24, 48)	35 (24, 48)	34 (25, 46)	0.978
Total bilirubin (μmol/L, IQR)	8.2 (6.0, 11.4)	7.9 (5.9, 10.9)	8.5 (6.2, 11.6)	0.220
Creatinine (μmol/L, IQR)	63.1 (53.1, 73.3)	63.2 (53.6, 74.0)	63.1 (52.3, 72.1)	0.689
BUN (mmol/L, IQR)	4.6 (3.5, 6.1)	4.8 (3.6, 6.4)	4.6 (3.3, 5.9)	0.245
PaO_2_, (kPa, IQR)	61.0 (53.0, 66.0)	62.0 (53.0, 67.0)	59.0 (52.8, 65.0)	0.118
Beta-1,3-glucan (pg/ml, IQR)	254.5 (99.2, 596.7)	252.1 (98.7, 606.7)	256.1 (98.7, 568.9)	0.847
CD4+ T-cell count (cells/μL, IQR)	24.0 (13.0, 44.8)	22.8 (13.0, 44.3)	24.5 (13.0, 45.5)	0.653
CD4/CD8 ratio (IQR)	0.08 (0.05, 0.14)	0.07 (0.04, 0.14)	0.09, (0.05, 0.14)	0.288
HIV RNA (log_10_ copies/mL, IQR)	5.6 (5.3, 6.0)	5.6 (5.2, 6.0)	5.6 (5.3, 6.0)	0.424
*Treatment strategy*				
ART regimens containing INSTIs, n (%)	148 (40.8)	71 (42.0)	77 (39.7)	0.732

BMI, body mass index; AST, aspartate aminotransferase; ALT, alanine aminotransferase; BUN, blood urea nitrogen; PaO_2_, partial pressure of arterial oxygen

### Baseline

**Fig. 2:**
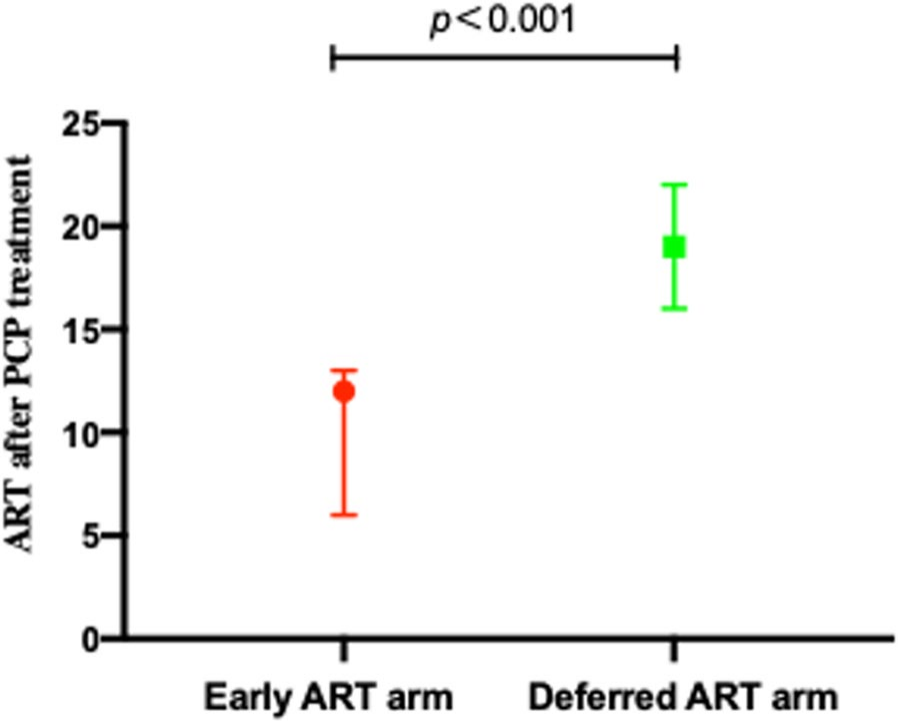
The duration (in days) between PCP treatment and ART initiation, median with IQR

Participants were primarily men (81.5%), with a median age of 47 years. The main ethnic group was Han Chinese (95.0%). Thirty-nine percent of participants were smokers, and 25.9% used alcohol. The median BMI was 20.2 kg/m^2^ [IQR, 18.7–22.5 kg/m^2^]. The median respiratory rate and body temperature were 22 breaths/min [IQR, 20–25] and 37.0℃ [IQR, 36.5–38.0], respectively. Routine blood tests, and liver and kidney function testing results at baseline showed no significant differences between the two study arms, with the exception of red blood cell counts. The median PaO_2_ was 61.0 kPa [IQR, 53.0–66.0]. The median beta-1,3-glucan level was 254.5 pg/ml [IQR, 99.2–596.7]. Participants had a median CD4+ T-cell count of 24.0 cells/μL [IQR, 13.0–44.8], a median CD4/CD8 ratio of 0.08 [IQR, 0.05–0.14], and a median log_10_ HIV viral RNA load of 5.6 [IQR, 5.3–6.0], as shown in Table 1. Integrase inhibitors (INSTIs) were used in 42.0% (71/169) of participants in the early ART arm and 39.7% (77/194) of participants in the deferred ART arm. The early and deferred arms started ART a median of 12 (6, 13) and 19 (16, 22) days after start of PCP treatment, respectively, as shown in Fig. 2.

### Primary outcomes

Adjusted for age, smoking, and red blood cell counts at baseline, there were no significant differences for any primary outcome between the two study arms, which evaluated the rates of death from any cause, and AIDS-defining events. In total, 46 participants (12.7%) succumbed; 20 (11.8%) in the Early ART arm, and 26 (13.4%) in the Deferred ART arm (*p* = 0.860). Seventeen participants (10.1%) in the Early ART arm, and 26 (13.4%) in the Deferred ART arm (*p* = 0.412) developed AIDS-defining events. However, a difference in the rates of patients who died before ART was initiated was observed between the two treatment arms. Eleven participants (55.0%) in the early ART arm, and 23 participants (88.5%) in the Deferred ART arm (*p* = 0.026) died before ART had ever been started, as shown in Table 2.

**Table 2:** Outcomes in the two study arms

Outcomes	Early ART arm	Deferred ART arm	*p* value
All-cause mortality	11.8% (20/169)	13.4% (26/194)	0.860^a^
Number of patients in the deceased population who did not initiate ART	55.0% (11/20)	88.5% (23/26)	0.026
AIDS-defining events	10.1% (17/169)	13.4% (26/194)	0.412
Patients admitted to ICU	1.8% (3/169)	1.5% (3/194)	1.000
Requirement for intubation	1.2% (2/169)	0.5% (1/194)	0.904
IRIS	1.2% (2/169)	1.5% (3/194)	1.000
Adverse events	41.4% (70/169)	37.1% (72/194)	0.465
Adverse events grade 3 or 4	16.6% (28/169)	17.5% (34/194)	0.919
Virological suppression at week 24	67.4% (58/86)	56.5% (61/108)	0.159
Virological suppression at week 48	81.2% (82/101)	81.5% (88/108)	1.000

ICU, intensive care unit; IRIS, immune reconstitution inflammatory syndrome

^a^Adjusted for age, smoking, red blood cell count at baseline

### Overall survival

**Fig. 3:**
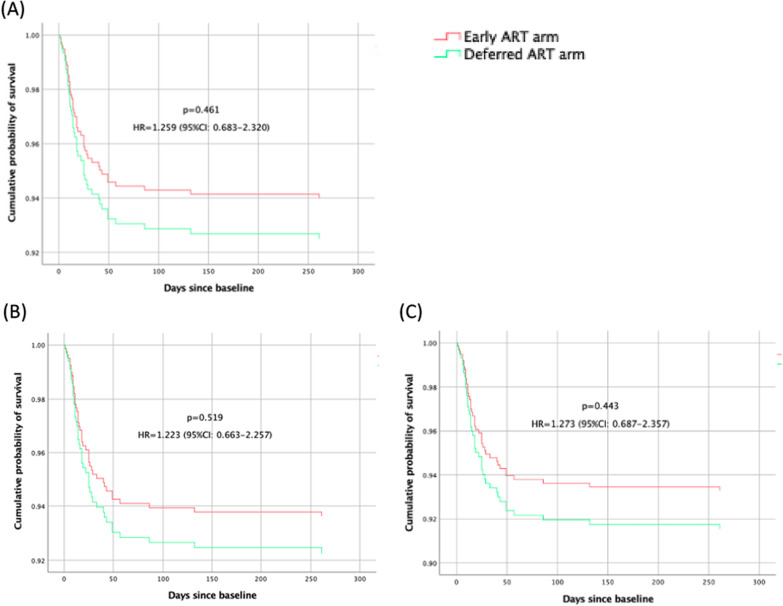
Kaplan–Meier curves depicting overall survival among all 363 participants in the two study arms (**A**), 351 participants with CD4+ T-cell counts less than 200 cells/μL (**B**), and 333 participants with CD4+ T-cell counts less than 100 cells/μL (**C**)

Mortality was influenced by age, respiration, temperature, AST, and ART regimens containing INSTIs via analysis by the Kaplan–Meier method and the Cox proportional-hazards model. Upon statistical analysis, adjusted for age, respiration, temperature, AST, and ART regimens containing INSTIs which had an effect on mortality, there was no significant difference in the 48-week time distribution of survival found between the two study arms (*p* = 0.461), as shown in Fig. 3A. For participants with CD4+ T-cell count less than 200 cells/μL, and also for those with CD4+ T-cell count less than 100 cells/µL, there was no significant difference in the 48-week time distribution of survival found between the two treatment arms (*p* = 0.519), as shown in Fig. 3B, C.

### Secondary outcomes

Two of 169 participants in the Early ART arm and three of 194 participants in the Deferred ART arm developed paradoxical PCP/IRIS (*p* = 1.000). Three participants (1.8%) in the Early ART arm compared to three participants (1.5%) in the Deferred ART arm (*p* = 1.000) were admitted to ICU. Two participants (1.2%) in the Early ART arm required intubation, compared to one participant (0.5%) in the Deferred ART arm (*p* = 0.904) (Table 2). We observed no difference in the total number of adverse events between the two arms, i.e., adverse events occurred in 70 of 169 participants in the Early ART arm, and in 72 of 194 participants in the Deferred ART arm (*p* = 0.465). Adverse events of grade 3–4 were reported in 28 of the 169 participants in the Early ART arm and 34 of 194 participants in the Deferred ART arm (*p* = 0.919), as shown in Table 2.

### Median change in immune indices from baseline to week 48

**Fig. 4:**
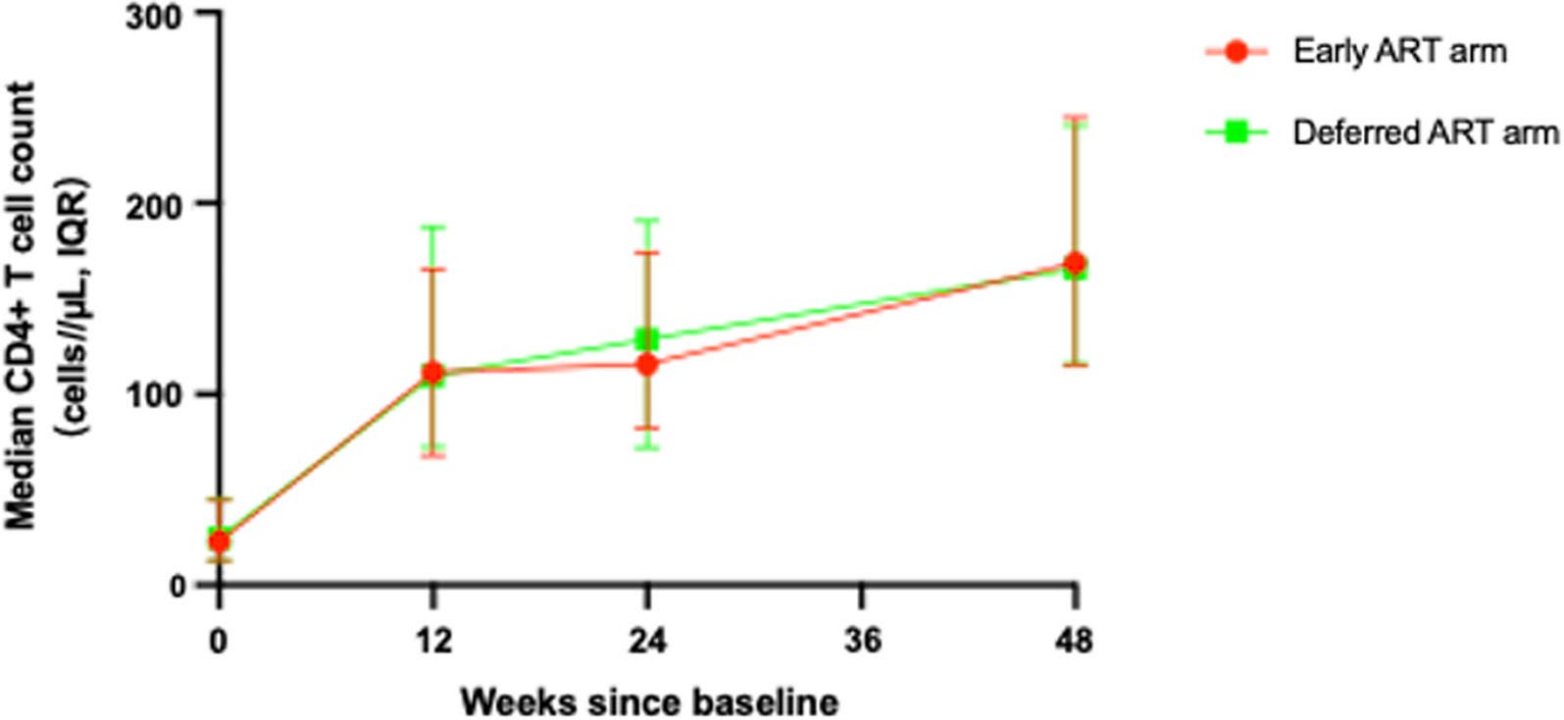
Median change in CD4+ T-cell counts from baseline to week 48

Median CD4+ T-cell counts increased in both study arms. We recorded no significant differences in changes to CD4+ T-cell counts between the two study arms from baseline to week 48, as shown in Fig. 4.

### Virological suppression

At week 24, there were 86 participants in the Early ART initiation arm and 108 participants in the Deferred ART initiation arm for the analysis of virological suppression, and the results showed that 67.4% (58/86) of participants in the Early ART initiation arm and 56.5% (61/108) participants in the Deferred ART initiation arm achieved virological suppression (*p* = 0.159). At week 48, a total of 209 participants presented for HIV RNA testing (101 in the Early ART initiation arm and 108 in the Deferred ART initiation arm), and 81.2% (82/101) of participants in the Early ART initiation arm and 81.5% (88/108) of participants in the Deferred ART initiation arm achieved virological suppression (*p* = 1.000), as shown in Table 2.

## Discussion

In order to further prioritize therapeutic strategies and reduce mortality, we conducted the present trial which aimed to investigate the optimal timing for ART initiation in AIDS patients with moderate to severe PCP in China. The mortality observed in the present study was 12.7% (46/363). The major strength of our study is that it provides important data for the salient question as to when to initiate ART in the extremely vulnerable group of HIV-infected patients with moderate to severe PCP.

In concordance with one previous study [10], we noted no differences in median CD4+ T-cell counts between the two study arms of our investigation. A prospective study which enrolled a total of 61 patients, conducted by Schäfer et al., in AIDS patients with toxoplasmosis and PCP observed that there was no significant difference in immunological outcomes between these two cohorts [10]. There was no statistically significant difference in both treatment arms in assessments of virological suppression at week 24 and 48. This is consistent with what usually occurs in acute OIs in HIV-infected patients, as previously reported [8].

Most adverse events in the present study were mild. Overall, the frequency of adverse events did not differ between the two study arms, and there was no evidence of a difference in effect between the study arms. In our study, Early ART initiation did not result in an increase in adverse events or serious adverse events, compared to the Deferred ART initiation arm.

IRIS was uncommon in the present study (1.4%) although participants had advanced immunodeficiency and had robust increases in CD4+ T-cell counts. The incidence of paradoxical IRIS in the present study was lower than that reported in other published studies [8, 11, 12]. Roade Tato et al., reported 6 cases of IRIS development in a cohort of 123 (4.9%) HIV-infected patients with PCP who started ART [11]. In a published study which enrolled a total of 282 patients with OIs, and including 177 AIDS/PCP patients, the reported incidence of IRIS after ART initiation was 7% [8]. Another prospective study conducted in South Africa observed that 10.4% of HIV-infected patients (44/423) developed IRIS [12]. It is not precisely clear why the rate of IRIS in our trial was as low as was observed. However, our results demonstrate that the risk of IRIS development should not be a reason to defer ART in AIDS/PCP patients.

In 2009, the AIDS Clinical Trials Group reported that AIDS progression and mortality in HIV-infected patients with non-tuberculous OIs are decreased if ART was initiated early (within 14 days of starting acute OI treatment) [8]. The preceding study observed that patients with OIs, including PCP, may be able to benefit from early ART initiation [8]. However, another recent study which enrolled 50 AIDS/PCP patients found that there were no significant differences in safety and efficacy between an Early ART initiation arm, and a Deferred ART initiation arm [10]. In concordance with the above study, we found that our Early ART arm had no increase in mortality and AIDS-defining events compared to our Deferred ART arm. These results support Early ART initiation in patients presenting with AIDS/PCP, should there be no major contraindications.

Latest guidelines recommend that ART should be started as soon as possible within 2 weeks of initiating tuberculosis treatment, regardless of CD4+ T-cell count [13]. However, one previous study recommends that tuberculosis patients initiate ART according to different levels of CD4+ T-cell count [14]. We chose to refer to previous tuberculosis guidelines in order to observe whether a different timing of ART initiation should be used for patients with different levels of CD + T-cell count [15]. In the present study, there was no significant difference between the two study arms when different levels of CD4+ T-cell count were considered (Fig. 3). Therefore, it seems that different levels of CD4+ T-cell count do not necessarily require a different timing of ART initiation for AIDS/PCP patients with varying CD4+ T-cell counts.

In the present study, 34 of 46 patients who succumbed (11 in the Early ART arm and 23 in the Deferred ART arm) never initiated ART. This can be interpreted as evidence of the potential benefit of the strategy of initiating ART early. Given these results, we recommend that AIDS/PCP patients should initiate ART early in the course of PCP treatment, should there be no major contraindications to doing so. Although there may be potential clinical challenges (e.g., drug-drug interactions, additive drug toxicities, etc.) in initiating ART early in AIDS/PCP patients, the likely benefit to such patients appears to be substantial.

There are a few limitations to the present study. Firstly, this trial was conducted in Chinese AIDS/PCP patients exclusively, and whether our results may be safely extrapolated to other population groups requires further study. Secondly, the diagnosis of PCP for most participants was a clinical diagnosis rather than a definitive diagnosis because prevailing hypoxia and dyspnea in some participants made bronchoscopic examination and bronchoalveolar lavage technically difficult and potentially hazardous to undertake; nevertheless, we adopted a unified clinical diagnostic standard for patients with moderate to severe PCP who could not undergo these procedures. Thirdly, a significant proportion of patients did not complete the 48-week follow-up and/or did not undergo HIV RNA viral load testing due to the prevailing COVID-19 epidemic, and this may have introduced a degree of bias to our results. Fourthly, since we were not able to obtain reliable information on all causes of deaths in this trial, we were unable to estimate or speculate on the effect of cause of death on the rate of deaths that were related only to PCP or AIDS. Fifth, we were unable to clearly distinguish which specific drugs caused specific adverse events, and we were thus unable to estimate the rate of adverse events that were directly related to either PCP therapy or to other drugs used.

## Conclusion

Early ART initiation does not increase mortality, AIDS-defining events, IRIS, adverse events, and immunological or virological outcomes in AIDS/PCP patients. These results support the early initiation of ART in patients with moderate to severe AIDS/PCP.

## Data Availability

The datasets used and analyzed during the present study are available from the corresponding author upon reasonable request.

## Abbreviations

AIDS: Acquired Immune Deficiency Syndrome
ALT: Alanine aminotransferase
ART: Antiretroviral therapy
AST: Aspartate aminotransferase
BMI: Body mass index
BUN: Blood urea nitrogen
HIV: Human immunodeficiency virus
ICU: Intensive care unit
IRIS: Immune reconstitution inflammatory syndrome
OI: Opportunistic infection
PaO_2_: Partial pressure of arterial oxygen
PCP: Pneumocystis pneumonia
RBC: Red blood cells
TMP-SMZ: Trimethoprim-sulfamethoxazole
UNL: Upper normal limit
WBC: White blood cells
